# Aprotinin Inhibits Thrombin Generation by Inhibition of the Intrinsic Pathway, but is not a Direct Thrombin Inhibitor

**DOI:** 10.1055/s-0041-1735154

**Published:** 2021-08-31

**Authors:** Ton Lisman, Jelle Adelmeijer, Dana Huskens, Joost C. M. Meijers

**Affiliations:** 1 Surgical Research Laboratory, Department of Surgery, University of Groningen, University Medical Center Groningen, Groningen, The Netherlands; 2 Department of Biochemistry, Cardiovascular Research Institute Maastricht (CARIM), Maastricht University, Synapse Research Institute, Maastricht, The Netherlands; 3 Department of Molecular Hematology, Sanquin Research, Amsterdam, The Netherlands; 4 Department of Experimental Vascular Medicine, Amsterdam UMC, University of Amsterdam, Amsterdam, The Netherlands

**Keywords:** fibrinolysis, aprotinin, tranexamic acid, thrombin, platelets

## Abstract

**Background** Aprotinin is a broad-acting serine protease inhibitor that has been clinically used to prevent blood loss during major surgical procedures including cardiac surgery and liver transplantation. The prohemostatic properties of aprotinin likely are related to its antifibrinolytic effects, but other mechanisms including preservation of platelet function have been proposed.

**Aim** Here we assessed effects of aprotinin on various hemostatic pathways in vitro, and compared effects to tranexamic acid(TXA), which is an antifibrinolytic but not a serine protease inhibitor.

**Methods** We used plasma-based clot lysis assays, clotting assays in whole blood, plasma, and using purified proteins, and platelet activation assays to which aprotinin or TXA were added in pharmacological concentrations.

**Results** Aprotinin and TXA dose-dependently inhibited fibrinolysis in plasma. Aprotinin inhibited clot formation and thrombin generation initiated via the intrinsic pathway, but had no effect on reactions initiated by tissue factor. However, in the presence of thrombomodulin, aprotinin enhanced thrombin generation in reactions started by tissue factor. TXA had no effect on coagulation. Aprotinin did not inhibit thrombin, only weakly inhibited the TF-VIIa complex and had no effect on platelet activation and aggregation by various agonists including thrombin. Aprotinin and TXA inhibited plasmin-induced platelet activation.

**Conclusion** Pharmacologically relevant concentrations of aprotinin inhibit coagulation initiated via the intrinsic pathway. The antifibrinolytic activity of aprotinin likely explains the prohemostatic effects of aprotinin during surgical procedures. The anticoagulant properties may be beneficial during surgical procedures in which pathological activation of the intrinsic pathway, for example by extracorporeal circuits, occurs.

## Introduction

Aprotinin is a broad acting serine protease inhibitor that has been clinically used to prevent blood loss during major surgical procedures including cardiac surgery and liver transplantation. 1 2 The drug was withdrawn from clinical use in 2007, following a randomized controlled clinical trial that suggested aprotinin use in cardiac surgery was associated with an increased risk of complications and death. 3 The comparator in these studies was a lysine analogue (either tranexamic acid or epsilon aminocaproic acid).

The effect of aprotinin in reducing perioperative blood loss is generally assumed to be related to its antifibrinolytic effect. 4 Aprotinin is a direct inhibitor of plasmin, but also inhibits FXIIa-dependent activation of fibrinolysis by inhibiting kallikrein which activates factor XII in the contact pathway. By inhibition of kallikrein, aprotinin also inhibits activation of coagulation via the contact pathway. Finally, aprotinin has anti-inflammatory effects related to inhibition of kallikrein. A combined antifibrinolytic, anticoagulant, and anti-inflammatory effect of aprotinin is clinically evident by decreased D-dimer, prothrombin fragment 1 + 2, and bradykinin plasma levels in patients undergoing cardiac surgery receiving aprotinin compared with those who did not. 5 6 7 8 9 The antifibrinolytic effects of aprotinin may also reduce plasmin-mediated activation of platelets 10 11 or plasmin-mediated proteolysis of platelet receptors such as glycoprotein Ib, 12 thereby preserving platelet function.

As aprotinin is a broad-spectrum serine protease inhibitor, other targets that may explain the mode of action of aprotinin as a hemostatic drug have been proposed. It has even been questioned whether the antifibrinolytic effect is a main contributor to the prohemostatic effect of aprotinin. 13 Other mechanisms that have been proposed to contribute to the prohemostatic properties of aprotinin are plasmin-independent platelet-preserving effects and anticoagulant properties, although it is not entirely clear how anticoagulant properties could contribute to a prohemostatic effect. Conflicting reports on these alternative modes of action of aprotinin have appeared in literature. For example, whereas some studies have reported that aprotinin inhibits platelet activation by a wide variety of agonists, 14 15 16 other studies report that aprotinin inhibits platelet activation by thrombin but not by other agonists. 17 18 Specifically, studies from one laboratory have shown that aprotinin in clinically relevant concentrations inhibits thrombin-mediated platelet activation via protease activated receptor (PAR)-1, but not via PAR-4. 17 19 This selective inhibition of PAR-1 activation is not explained by direct thrombin inhibition of aprotinin, which would also block PAR-4 activation. In addition, one study has demonstrated that aprotinin is a direct thrombin inhibitor, 18 but the K _i_ reported (61 uM or ∼2900 KIU/ml) is around an order of magnitude higher than peak plasma concentrations that are clinically observed (Plasma concentrations of aprotinin in patients undergoing cardiac or liver transplant surgery are between 100 and 400 KIU 20 21 22 23 ). Clinically, substantial evidence for a platelet-preserving effect of aprotinin exists, 24 25 26 27 and although these platelet-preserving effects have been suggested to be mediated by direct effects of aprotinin on platelets, 28 the effect may also be fully explained by a combination of inhibition of plasmin-mediated platelet activation 10 and cleavage of platelet receptors, 12 and a decrease of thrombin generation via the intrinsic pathway. 7 Also, it has been suggested that aprotinin is an inhibitor of the tissue factor-FVIIa complex, 29 although the aprotinin doses used in this study were supratherapeutic.

To assess effects of aprotinin in clinically relevant concentrations on the hemostatic system, we have studied the effects of aprotinin in a range of in vitro assays using the currently marketed clinical product. Results were compared with tranexamic acid, which is an antifibrinolytic drug that is thought to reduce bleeding only by reducing plasmin formation.

## Materials and Methods

### Materials

Aprotinin was a generous gift from Nordic Pharma (Baarn, The Netherlands), tranexamic acid was from Mylan B.V. (Amstelveen, The Netherlands), Dabigatran was purchased from Alsachim (Illkirch Graffenstaden, France), recombinant tissue factor pathway inhibitor (TFPI) was from R&D systems (Minneapolis, MI, USA). Pooled normal plasma was obtained by combining citrated plasma from >200 healthy volunteers.

### Clot Lysis Assay

Fibrinolytic capacity in plasma was assessed by monitoring changes in turbidity during clot formation and lysis of a tissue factor–induced clot by exogenous tissue plasminogen activator, as described previously. 30 Clot lysis time was defined as the time from the midpoint of the clear to maximum turbid transition, representing clot formation, to the midpoint of the maximum turbid to the clear transition, representing clot lysis (a typical example of a clot lysis curve can be found here 31 ).

### PT/APTT

Prothrombin time (PT) and activated partial thromboplastin time (APTT) assays were performed on an automated coagulation analyzer (STACompact 3, Stago, Breda, the Netherlands) with the use of reagents and protocols from the manufacturer.

### Thrombin Generation Test

Thrombin generation assays were performed with the fluorimetric method calibrated automated thrombinography, as described previously by Hemker et al. 32 Coagulation was activated using a diluted PT reagent (Innovin, Siemens, Erlangen, Germany, 1/900 final dilution), or a diluted APTT reagent (Synthasil, Werfen, Breda, The Netherlands, 1/40 dilution).The endogenous thrombin potential was derived with software from Thrombinoscope (Maastricht, the Netherlands). In selected experiments, thrombin generation was performed in the presence of soluble thrombomodulin (10 nM final concentration, Synapse, Maastricht, The Netherlands).

### Viscoelastic Test

Whole blood clot formation was assessed with the ClotPro analyser (Nodia, Breda, the Netherlands), a new generation viscoelastic test that is very similar to TEG and ROTEM ( www.clot.pro ). Clotting was initiated by commercially available reagents (Ex test and In test) for the extrinsic and intrinsic pathway.

### Chromogenic Assays

In a 96-well plate, thrombin (Enzyme Research Laboratories, South Bend, IN, USA, 1 U/ml) was mixed with Biophen Thrombin chromogenic substrate (Nodia, 1 mM) in Tris-buffered saline. Immediately after mixing, optical density at 405 nm was read every 10 seconds for 3 minutes in a VERSAmax reader (Molecular devices, San Jose, CA, USA) at 37°Celcius. The initial slope of the time vs optical density curve reflects the amidolytic activity of thrombin. Similarly, the activity of recombinant factor XIa (purified as described, 33 10 nM), factor XIIa (Enzyme Research Laboratories, South Bend, IN, 19,5 µM), kallikrein (Stago, 2 nM), and activated protein C (a generous gift from the late Dr. Walter Kisiel, University of Albuquerque, New Mexico, 10 nM) were measured toward S2302 (kallikrein and FXIIa) or S2366 (XIa and activated protein C) at a final concentration of 0.5 mM (Chromogenix, Molndal, Sweden). The initial slope of the reactions without inhibitory compounds present was set at 100.

### Tissue Factor-factor VIIa-mediated Activation of Factor X

In a 96-well plate, tissue factor (Innovin, 1/40 final dilution), was mixed with recombinant factor VIIa (Novo Nordisk, Bagsvaerd, Denmark, 20 ng/ml) in Tris-buffered saline. Factor X was added (Bioconnect, the Netherlands, 10 ug/ml) and incubated for 3 minutes at 37°Celcius. Next, Pefachrome Xa (Stago, 1 mM) was added immediately thereafter, optical density at 342 nm was read every 10 seconds for 20 minutes in a VERSAmax reader at 37°Celcius. The initial slope of the time vs optical density curve reflects the amount of factor Xa generated. The initial slope of the reactions without inhibitory compounds present was set at 100.

### Fibrin Generation

In a 96-well plate, thrombin (1 U/ml) was mixed with fibrinogen (Nodia, 2 mg/ml) in Tris-buffered saline. Immediately after mixing, optical density at 340 nm was read every 10 seconds for 20 minutes in a VERSAmax reader. Clotting time was defined as the time from mixing to the midpoint of the clear to maximum turbid transition.

### Platelet Aggregation

Platelet-rich plasma was prepared by a centrifugation of human whole blood at 180 g for 10 minutes. Washed platelets were prepared as described previously. 34 Platelet-rich plasma was stimulated with collagen (Stago, Breda, The Netherlands, 2 μg/ml). Washed platelets were stimulated by thrombin (1 U/ml) in the presence of GPRP peptide (Sigma-Aldrich,1 mM). Aggregation was monitored using a Chrono-Log Model 700 (Chronolog, Havertown, PA, USA).

### Platelet Activation

Platelet activation in whole blood was studied by flow cytometry as described previously. 35 In short, whole blood was diluted 1:3 with HEPES-buffered saline (HBS, 10 mmol/l HEPES, 150 mmol/l NaCl, 1 mmol/l MgSO4, 5 mmol/l KCl, pH 7.4). From this diluted blood, 5 μl was added to each reaction mixture with a total volume of 20 μl. In this reaction mixture, 2 μl FITC-conjugated PAC-1, 1.5 μl PE-conjugated anti-P-selectin and 0.5 μl APC-conjugated anti-CD42b was present. In addition, reaction mixtures contained the protease activated receptor (PAR)-1 agonist thrombin receptor activator peptide (TRAP-6 (SFLLRN), H-2936; Bachem, Germany, 30 μM final concentration), the glycoprotein VI (GPVI) agonist collagen-related peptide (CRP, purchased from Professor Farndale, University of Cambridge, UK, 5 μg/ml final concentration), the P2Y _12_ agonist 2MeS-ADP (1624, Tocris, 2μM final concentration), thrombin (2 or 10 nM in the presence of 1 mM GPRP peptide, final concentrations), or plasmin (100 μg/ml). The reaction mixtures were incubated for exactly 20 min at 37 °C. Reactions were stopped by adding 250 μl fixation solution (137 mmol/l NaCl, 2.7 mmol/l KCl, 1.12 mmol/l NaH _2_ PO _4_ , 1.15 mmol/l KH _2_ PO _4_ , 10.2 mmol/l Na _2_ HPO _4_ , 4 mmol/l EDTA, 0.5% formaldehyde). An Accuri C6 (BD Biosciences, Erembodegem, Belgium) was used to analyze the samples. First, using the forward and sideward scatter pattern and gating on the CD42b positive cells, platelets were discriminated from other cells. Fluorescent intensity in the FITC gate and PE channels were determined and results are expressed as median fluorescent intensity (MFI) corrected for the MFI of blood to which no platelet agonist was added.

## Results

### Aprotinin and Tranexamic Acid Inhibit Fibrinolysis in Plasma

**Fig. 1:**
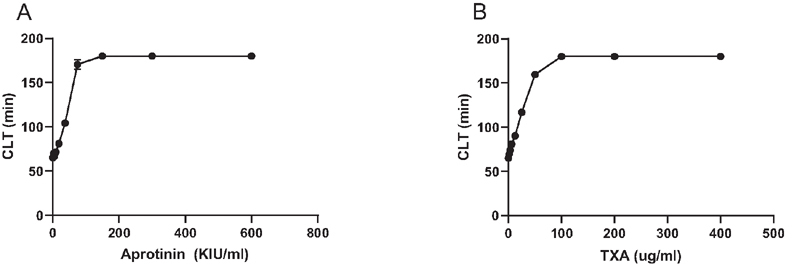
The antifibrinolytic effect of aprotinin and tranexamic acid (TXA). Aprotinin or TXA were added to pooled normal plasma in increasing concentrations and clot lysis times were determined. Shown are mean values of 3 independent experiments. Error bars indicate standard deviations (but are not visible for the majority of data points as standard deviations were very low).

We first confirmed aprotinin and tranexamic acid to have potent and dose-dependent antifibrinolytic effects in vitro at clinically relevant concentrations. Aprotinin or tranexamic acid were added to pooled normal plasma which was subsequently tested in a plasma clot lysis assay. Both aprotinin and tranexamic acid dose-dependently increased clot lysis time, with no detectable lysis during the three hours the assay is run at the highest doses tested ( Fig. 1 ). Aprotinin plasma levels between 100 and 400 KIU/ml 20 21 22 23 and TXA plasma levels between 30 and 100 ug/ml 36 37 have previously been measured in patients undergoing cardiac surgery or liver transplantation.

### Aprotinin Inhibits Coagulation Activated by the Intrinsic but not the Extrinsic Pathway

**Fig. 2:**
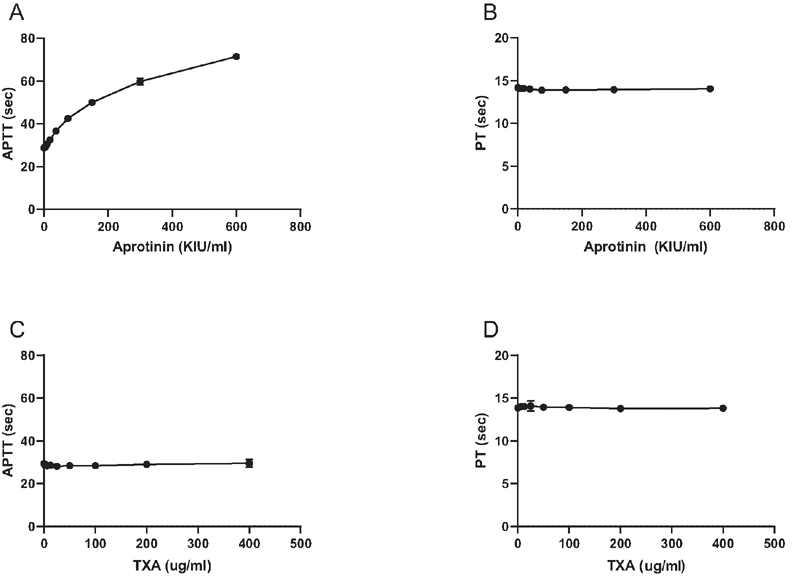
Effects of aprotinin ( **A,B** ) and TXA ( **C,D** ) on the activated partial thromboplastin time (APTT, A,C) and prothrombin time (PT, B,D). Aprotinin and TXA were added to pooled normal plasma in increasing concentrations and APTT and PT assays were performed. Shown are mean values of 3 independent experiments. Error bars indicate standard deviations (but are not visible for the majority of data points as standard deviations were very low).

**Fig. 3:**
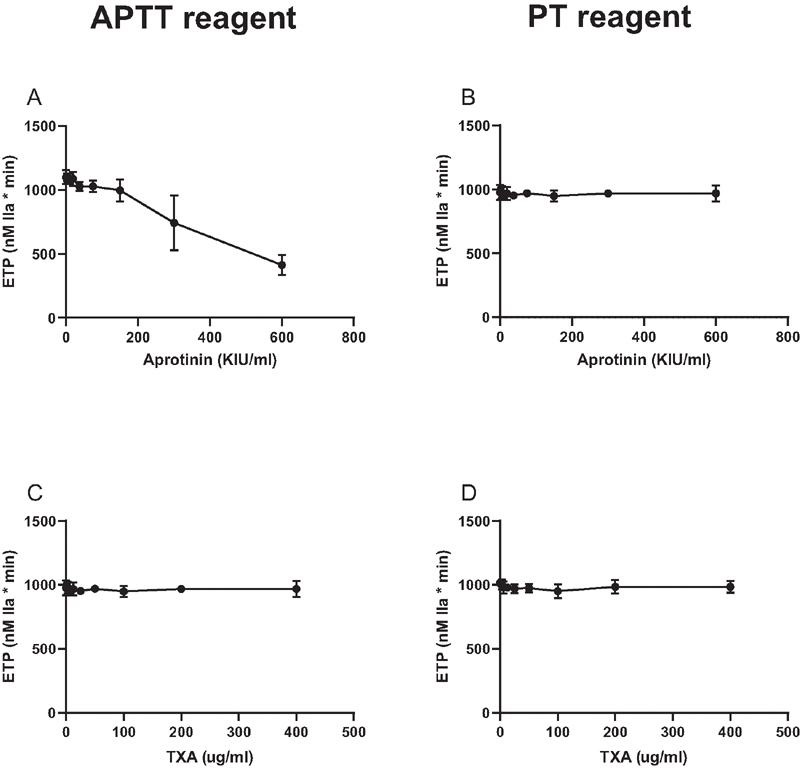
Effects of aprotinin and TXA on the Endogenous Thrombin Potential (ETP). Aprotinin and TXA were added to pooled normal plasma in increasing concentrations and samples were subjected to calibrated automated thrombinography. Coagulation was initiated by a dilute APTT reagent ( **A** and **C** ) or a dilute PT reagent ( **B** and **D** ). Shown are mean ETP values derived from thrombin generation curves of 3 independent experiments. Error bars indicate standard deviations.

**Fig. 4:**
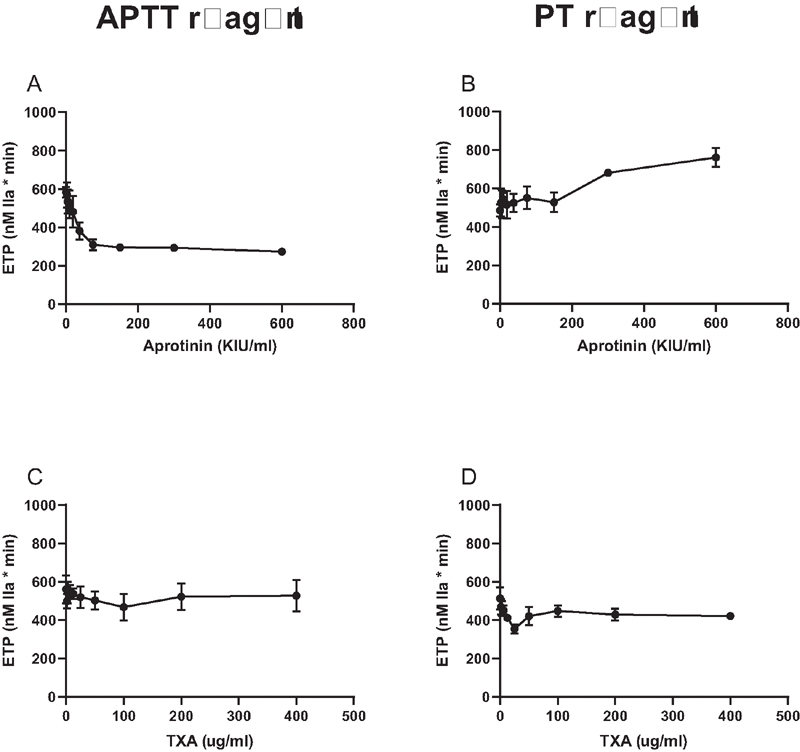
Effects of aprotinin and TXA on the Endogenous Thrombin Potential (ETP) in the presence of soluble thrombomodulin. Aprotinin and TXA were added to pooled normal plasma in increasing concentrations and samples were subjected to thrombomodulin-modified calibrated automated thrombinography. Coagulation was initiated by a dilute APTT reagent ( **A** and **C** ) or a dilute PT reagent ( **B** and **D** ). Shown are mean ETP values derived from thrombin generation curves of 3 independent experiments. Error bars indicate standard deviations.

**Fig. 5:**
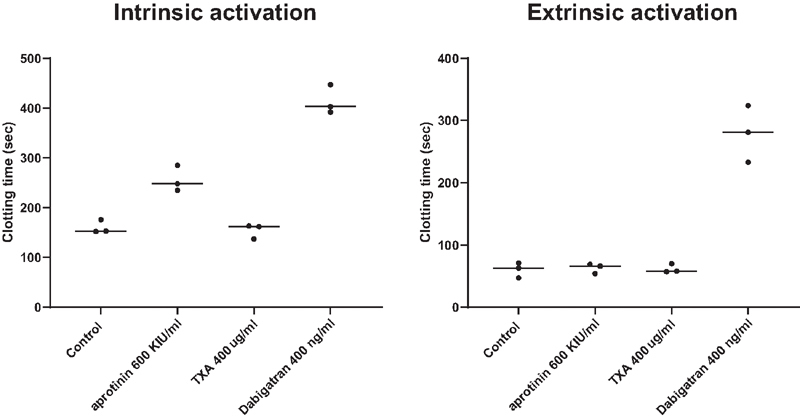
Effects of aprotinin, TXA, and dabigatran on whole blood clot formation following intrinsic or extrinsic activation of coagulation. Aprotinin or TXA were added to whole blood from healthy individuals and clot formation was monitored in a ClotPro device following activation by the Ex test (panel A) or In test (panel B). Clot times of three donors are shown. Horizontal bars indicate medians.

**Fig. 6:**
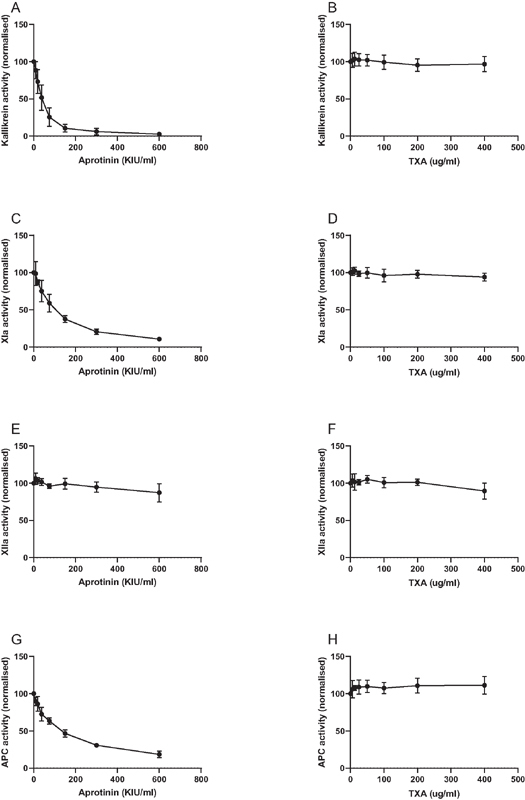
Effects of aprotinin and TXA on kallikrein, factor XIa, factor XIIa, and activated protein C. Aprotinin or TXA were added in increasing concentrations to purified activated coagulation factors, after which enzyme activities were determined using a chromogenic substrate. Activity levels in the absence of aprotinin or TXA were set at 100. Shown are mean values of 3 independent experiments performed in duplicate.

Aprotinin and tranexamic acid were added to pooled normal plasma and plasma samples were tested in PT and APTT assays. Aprotinin dose-dependently prolonged the APTT but had no effect on the PT ( Fig. 2A, B ). Tranexamic acid had no effect on both PT and APTT ( Fig. 2C, D ). Next, we performed thrombin generation tests in which coagulation was initiated by an APTT or a PT reagent. Aprotinin prolonged the lag time (data not shown) and decreased the ETP of a reaction initiated by an APTT reagent, but had no effect on thrombin generation when coagulation was initiated by a PT reagent ( Fig. 3A-B ). Tranexamic acid had no effect on thrombin generation regardless of the initiating trigger ( Fig. 3C-D ). However, when thrombin generation experiments were performed in the presence of soluble thrombomodulin, aprotinin still inhibited thrombin generation initiated by an APTT reagent, but stimulated thrombin generation initiated by a PT reagent ( Fig. 4 ). In a whole blood viscoelastic test, clot formation was inhibited by aprotinin only when clot formation was initiated by an activator of the intrinsic pathway ( Fig. 5 ). Aprotinin and tranexamic acid had no effect on maximal clot amplitude (data not shown). Aprotinin dose-dependently inhibited the activity of purified kallikrein and FXIa, but had limited effect on FXIIa. In addition, aprotinin dose-dependently inhibited activated protein C. Tranexamic acid did not inhibit kallikrein, FXIa, FXIIa, or activated protein C ( Fig. 6 ).

### Aprotinin is not an Inhibitor of Thrombin and only Weakly Inhibits the Tissue Factor-factor VIIa Complex

**Fig. 7:**
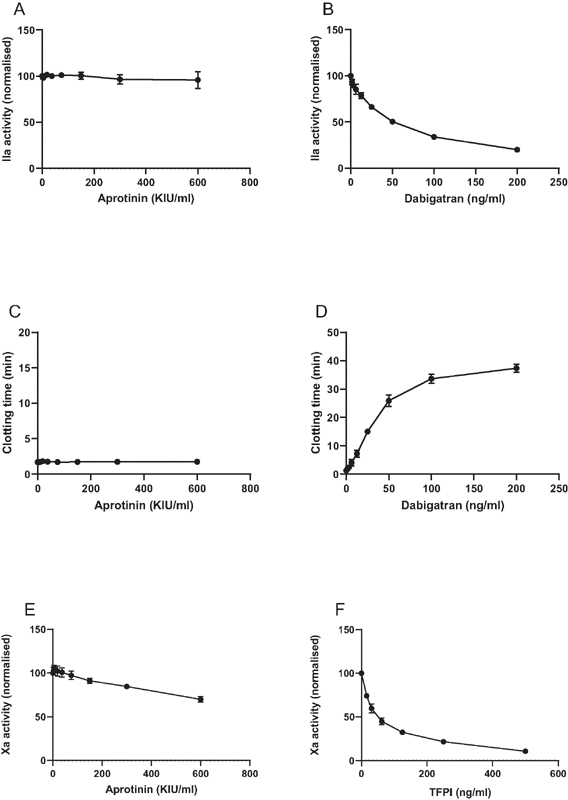
Effects of aprotinin on thrombin and the tissue factor-factor VIIa complex. **A** and **B** : Aprotinin or dabigatran were added in increasing concentrations to purified thrombin after which thrombin activity was assessed using the chromogenic substrate S2238. **C** and **D** : aprotinin and dabigatran were added in increasing concentrations to purified fibrinogen, after which thrombin was added. Clot formation was monitored by turbidity measurements over time. **E** and **F** : Aprotinin or TFPI were added to the tissue factor-factor VIIa complex in the presence of factor X and factor Xa generation over time was assessed using a chromogenic substrate. Shown are mean values of 3 independent experiments. Error bars indicate standard deviations (but are not visible for the majority of data points as standard deviations were very low).

We assessed the potential inhibitory effect of aprotinin on purified thrombin. First, we added aprotinin or dabigatran to thrombin in the presence of the chromogenic substrate S2238. Whereas aprotinin had no effect on the conversion of the chromogenic substrate by thrombin, dabigatran dose-dependently inhibited the activity of thrombin toward the chromogenic substrate ( Fig. 7A, B ). Similarly, when thrombin was added to purified fibrinogen, aprotinin had no effect on clot formation, whereas dabigatran dose-dependently delayed clot formation ( Fig. 7C, D ).

When aprotinin was added to a mixture of tissue factor, factor VIIa, and factor X, a slight decrease of FXa generation was observed at high aprotinin concentrations, whereas TFPI dose-dependently inhibited Xa generation (7E/F).

### Aprotinin does not Inhibit Platelet Aggregation Induced by Thrombin or Collagen

**Fig. 8:**
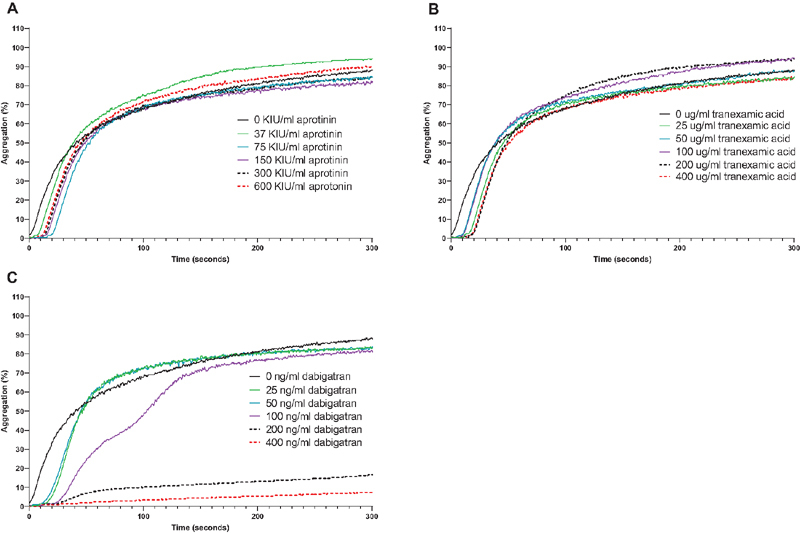
Effects of aprotinin and TXA on thrombin-induced platelet aggregation. Aprotinin (panel A), tranexamic acid (panel B), or dabigatran (panel C) were added in increasing concentrations to washed human platelets after which platelet aggregation was initiated by addition of 1 U/ml of thrombin. Shown is a representative example of experiments performed with three different platelet donors.

**Fig. 9:**
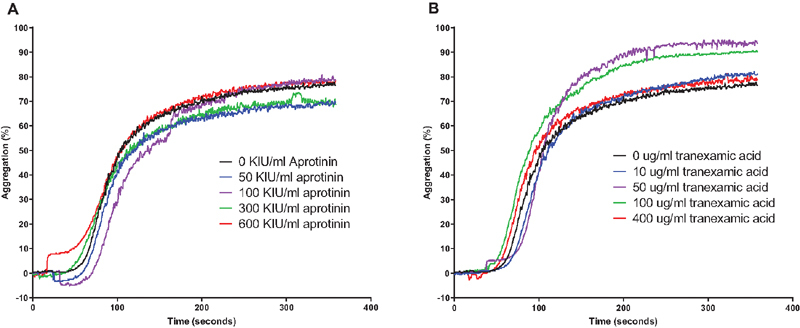
Effects of aprotinin and TXA on collagen-induced platelet aggregation. Aprotinin (panel A), or tranexamic acid (panel B) were added in increasing concentrations to human platelet-rich plasma after which platelet aggregation was initiated by addition of 2 ug/ml of collagen. Shown is a representative example of experiments performed with three different donors.

Washed platelets were stimulated with thrombin in the presence of GPRP, which prevents fibrin formation, and aggregation was monitored over time. As shown in Fig. 8A, B, C , aprotinin or tranexamic acid did not prevent platelet aggregation by thrombin, but dabigatran dose-dependently blocked aggregation. In platelet-rich plasma, aprotinin or TXA had no effect on collagen-induced aggregation ( Fig. 9A, B ).

### Aprotinin Inhibits Platelet Activation by Plasmin, but has no Effect on Platelet Activation by Various Agonists

**Fig. 10:**
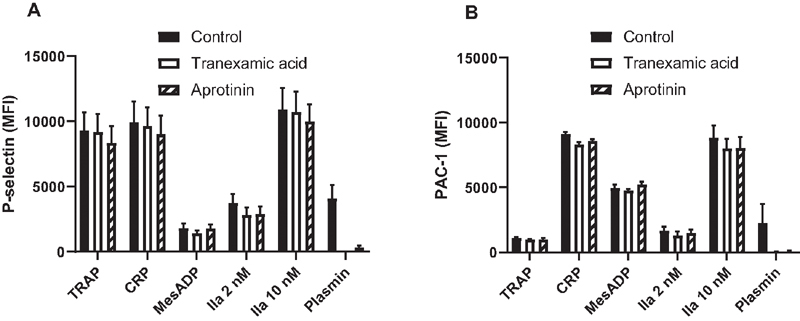
Effects of aprotinin and TXA on platelet activation by thrombin receptor activating peptide (TRAP), collagen-related peptide (CRP),2MeS-ADP, thrombin (IIa), and plasmin. Diluted whole blood was stimulated with one of the mentioned agonists or vehicle, and P-selectin expression (panel A) or activation of αIIbβ3 (panel B) were assessed by flow cytometry using anti-P-selectin or the PAC-1 antibody. Shown are median fluorescence intensity (MFI) corrected for values obtained in unstimulated samples. Bars indicate means of three independent experiments each performed in triplicate, error bars indicate standard deviations.

When whole blood was stimulated with TRAP, CRP, 2MeS-ADP, or thrombin, aprotinin (600 KIU/ml) or tranexamic acid (400 ug/ml) had no effect on platelet activation as assessed by platelet P-selectin expression ( Fig. 10A ) or PAC-1 binding ( Fig. 10B ), which reflects activation of integrin αIIbβ3. In contrast, both tranexamic acid and aprotinin fully blocked plasmin-induced platelet activation.

## Discussion

This study shows that aprotinin at clinically relevant concentrations has both antifibrinolytic and anticoagulant properties, and inhibits plasmin-induced platelet activation. The anticoagulant effect of aprotinin is restricted to inhibition of intrinsic activation mediated by inhibition of kallikrein and factor XIa. Under our experimental conditions, aprotinin did not inhibit thrombin, thrombin-mediated fibrinogen to fibrin conversion, or platelet activation by various agonists including thrombin, and only weakly inhibited the TF-VIIa complex. The inhibitory effect on the purified TF-VIIa complex is likely physiologically insignificant, as aprotinin did not inhibit TF-induced thrombin generation in plasma. Aprotinin increased thrombin generation via the extrinsic pathway in the presence of thrombomodulin, likely as a result of inhibition of activated protein C. 38 39 However, as aprotinin decreases thrombin generation in vivo, 5 6 7 it is unlikely this prohemostatic effect of aprotinin is clinically relevant. Collectively, our results suggest that plasmin and kallikrein are the only relevant hemostatic targets of aprotinin at clinically used concentrations in a plasma or whole blood environment. Tranexamic acid only had antifibrinolytic properties, and thus mechanistically, aprotinin has two theoretical advantages over tranexamic acid. First, the anti-inflammatory properties via inhibition of kallikrein may be beneficial. Second, the inhibitory effects of aprotinin on intrinsic activation of coagulation may mean that undesired activation of coagulation, for example by cardiopulmonary bypass circuits, is prevented by aprotinin, whereas physiological hemostasis remains intact. Aprotinin thus may function as an anticoagulant that is antithrombotic without substantially increasing bleeding risk, similar to new-generation antithrombotic agents that target FXI or FXII. 40

In our in vitro experiments, the antifibrinolytic and anticoagulant effects of aprotinin are apparent at plasma concentrations of 75 KIU and higher. As the plasma concentrations of aprotinin in vivo (in patients undergoing cardiac or liver transplant surgery) are between 100 and 400 KIU, 20 21 22 23 it is likely that the antifibrinolytic and anticoagulant effects of aprotinin described herein are clinically relevant. Indeed, aprotinin decreases plasma levels of D-dimer and prothrombin fragment 1 + 2 in patients undergoing cardiac surgery. 5 6 7 8 9

Our data are also in agreement with previous studies demonstrating in vivo platelet-preserving effects of aprotinin. 24 25 26 27 By inhibition of plasmin, aprotinin prevents or decreases plasmin-mediated proteolysis of platelet receptors and plasmin-mediated platelet activation. In addition, by inhibition of thrombin generation via the intrinsic pathway, aprotinin may also prevent platelet activation.

We do not confirm previous data on direct inhibitory effects of aprotinin on platelet activation, and do not find aprotinin to be a relevant thrombin inhibitor at the doses tested (up to 600 KIU/ml, which is supratherapeutic), which is in line with the K _i_ of 61 uM or 2900 KIU/ml previously reported. Why we fail to find inhibitory effects of aprotinin on thrombin-induced platelet activation, or on platelet activation induced by other agonists is not entirely clear. We thus have no clear explanation for the divergent results with previous studies but do note that 1) the high K _i_ for thrombin does not readily explain why aprotinin at clinically relevant concentrations would inhibit thrombin-mediated platelets activation and 2) previous studies on the inhibitory effect of aprotinin on the direct thrombin-independent platelet inhibitory properties also yielded contradictory results. 14 15 16 17 19

Taken together, our in vitro studies show that aprotinin has both antifibrinolytic and anticoagulant activity at clinically relevant concentrations. We propose that the antifibrinolytic activity is primarily responsible for the prohemostatic effects of aprotinin during surgical procedures. In addition, we propose that the anticoagulant properties may be beneficial in clinical situations in which substantial activation of the intrinsic pathway of coagulation occurs. As aprotinin is a selective inhibitor of intrinsic coagulation activation, it will dampen undesired coagulation activation by, for example extracorporeal circuits, without interfering with physiological hemostasis, and will therefore not contribute to perioperative bleeding. Aprotinin has now carefully been reintroduced, but may have clinical applications beyond complex cardiac surgery. For example, the anticoagulant properties of aprotinin may be exploited in clinically challenging situations in which activation of the intrinsic pathway leads to thrombotic events. Such scenarios include extracorporeal circuits used outside cardiac surgery, such as ECMO. As we have shown that aprotinin is not a direct thrombin or platelet inhibitor, aprotinin is not expected to increase bleeding risk in these scenarios.

## Notes

**What is Known**

- Aprotinin is a clinically used antifibrinolytic with an incompletely characterized mode of action
- Aprotinin is an inhibitor of plasmin but also inhibits kallikrein which leads to inhibition of intrinsically activated coagulation and of inflammation
- Other proposed targets of aprotinin are the tissue factor-VIIa complex, thrombin, platelets, and protease activated receptor 1 on platelets

**What this Study Adds**

- We studied hemostatic effects of aprotinin in various in vitro models
- Aprotinin inhibits fibrinolysis and the intrinsic pathway of coagulation, but does not inhibit platelet function, thrombin, or TF-VIIa
- The anticoagulant properties of aprotinin might be beneficial during surgeries using extracorporeal circuits
